# Cardiovascular disease risk factors are associated with conventional lipids and apolipoproteins in South African adults of African ancestry

**DOI:** 10.1186/s12944-025-02591-w

**Published:** 2025-05-15

**Authors:** Anri Vorster, Ruan Kruger, Catharina MC Mels, Yolandi Breet

**Affiliations:** 1 Hypertension in Africa Research Team (HART), North-West University, Potchefstroom, South Africa; 2 SAMRC Extramural Unit for Hypertension and Cardiovascular Disease, Faculty of Health Sciences, North-West University, Potchefstroom, South Africa

**Keywords:** Apolipoprotein, Lipid, Atherosclerosis, Cardiovascular disease risk factors

## Abstract

**Background:**

Although conventional lipids (high density lipoprotein cholesterol (HDLC), low density lipoprotein cholesterol (LDLC), total cholesterol (TC) and triglycerides (TG)) are therapeutic targets to manage and prevent atherosclerotic cardiovascular disease (CVD), apolipoprotein (Apo) levels have sparked interest for their potential to improve CVD risk prediction. This study explored the relationships of traditional CVD risk factors with conventional lipids, as well as ApoA1, ApoB and its ratio (ApoB: ApoA1) in South African adults of African ancestry.

**Methods:**

This study included 1697 adults (aged 29 to 94) from the Prospective Urban Rural Epidemiology (PURE) study. The CVD risk markers included body mass index (BMI), physical activity index, tobacco use, dietary fat intake, γ-glutamyl transferase (γGT) and glycated haemoglobin (HbA1C). Conventional lipids were measured in serum samples using standard methodology, while ApoA1 and ApoB were measured using a multiplex magnetic bead immunoassay.

**Results:**

Stratified into tertiles of conventional lipid and Apo levels, trends emerged across multiple CVD risk markers, including BMI, tobacco use, fat intake, γGT and HbA1C levels. Higher tertiles of LDLC, TC, TG, ApoB and ApoB: ApoA1, along with the lowest tertiles of HDLC and ApoA1 exhibited higher prevalence of Type II diabetes mellitus (all *p* ≤ 0.024) and overweight or obesity (all except for TC, *p* ≤ 0.024). HDLC was negatively associated and LDLC, TC, and TG were positively associated with BMI (all *p* < 0.001) and HbA1C (all except for TC, *p* ≤ 0.005). Similarly, ApoA1 associated negatively with BMI (β=-0.067 (-0.125; -0.010), *p* = 0.022) and HbA1C (β=-0.071 (-0.122; -0.020), *p* = 0.007), while ApoB associated positively with BMI (β = 0.168 (0.117; 0.218), *p* < 0.001). The ApoB: ApoA1 showed positive associations with BMI (β = 0.213 (0.163; 0.263), *p* < 0.001) and HbA1C (β = 0.123 (0.074; 0.172), *p* < 0.001).

**Conclusions:**

In South African adults of African ancestry, ApoA1, ApoB and ApoB: ApoA1 levels are associated with various established CVD risk markers and suggests that these apolipoproteins may provide additional mechanistic insights beyond the conventional lipids to understand the aetiology of early cardiometabolic disease development.

## Background

Cardiovascular disease (CVD) is a global health concern, particularly in developing countries [1]. In Sub-Saharan Africa, CVD accounts for approximately 13% of all deaths, representing 5.5% of global CVD mortality [2, 3]. In South Africa specifically, the Heart and Stroke Foundation reports that CVD is responsible for one in six deaths (17.3%) [4]. Although women generally exhibit a lower incidence of CVD compared to men, clinical evidence indicates women experience poorer prognosis and higher mortality rates following an acute cardiovascular event [5].

The INTERHEART study, which included data from 52 countries, ranked dyslipidaemia, smoking, psychosocial factors, excess body weight, hypertension, diet, physical inactivity, diabetes mellitus and alcohol consumption as the predominant modifiable risk factors for CVD [6]. The contribution of these risk factors to the development and progression of CVD may occur through the major underlying pathological mechanism known as atherosclerosis [7, 8].

Although conventional lipid levels are generally targeted to manage and prevent atherosclerotic CVD, apolipoprotein (Apo) levels have sparked interest for their potential to improve CVD risk prediction [9, 10]. Residual CVD risk remains evident even among statin users, and markers such as Apolipoprotein B (ApoB), alongside non-HDLC and remnant cholesterol, have been proposed as more precise indicators of this persistent risk [11]. Apos are mainly responsible for the distribution of lipids to various tissues and organs as well as the regulation of lipoprotein metabolism [12]. Apolipoprotein A1 (ApoA1) provides structure to anti-atherogenic high density lipoproteins and activates the uptake of free cholesterol by high density lipoproteins as part of the reverse cholesterol pathway [12, 13]. ApoB is generally described as the scaffolding protein that provides structure to the atherogenic low density lipoproteins [14], and acts as a ligand for low density lipoprotein receptors, initiating the release of free cholesterol within cells [14].

A study conducted in China reported that ApoB levels were found to be more closely linked to atherosclerosis and CVD compared to conventional lipid markers, including high density lipoprotein cholesterol (HDLC), low density lipoprotein cholesterol (LDLC), total cholesterol (TC) and triglycerides (TG) [15]. The ratio of ApoB: ApoA1, which expresses the balance of atherogenic and anti-atherogenic particles, is also considered to be potentially valuable to predict atherogenesis [16]. In addition, it was reported in a review paper that ApoB: ApoA1 was found to strongly associate with CVD in Asian countries, Iraq, Nigeria and Sweden [17].

In terms of CVD risk factors, BMI, dyslipidaemia and Type II diabetes mellitus (T2DM) were reported to positively associate with ApoB and ApoB: ApoA1 and negatively with ApoA1 among adolescents from Brazil and adults from China [16, 18]. An analysis of data from multiple countries revealed that smoking also associate negatively with ApoA1 and positively with ApoB and ApoB: ApoA1 [19]. Additionally, alcohol intake was associated with increased HDLC and ApoA1 levels in a dose-dependent manner among White adults [20].

South Africa is experiencing an increasing burden of CVD, notably in coronary heart disease and stroke [21], but limited studies focused on Apos in the South African context. We therefore aimed to explore the relationship between CVD risk factors and the extended lipid profile (including conventional lipids, ApoA1, ApoB and ApoB: ApoA1) in South African adults of African ancestry.

## Methods

### Study design and participants

This cross-sectional study is part of the large-scale international Prospective Urban and Rural Epidemiology (PURE) study. The study consists of a unique cohort aimed at investigating the health status of populations from 27 countries, including South Africa, and related changes in modifiable habits that may predispose or protect against CVD [22].

Baseline data for this study was collected in 2005 from 6000 potential households located among urban and rural residents of the North West Province in South Africa [23]. Households were randomly chosen, and participants were recruited in line with the approved inclusion and exclusion criteria [24]. Participants who self-reported prior cardiovascular events, acute illness, pregnancy, or lactation were excluded to avoid possible confounding. After excluding *n* = 313 participants with incomplete conventional lipid and Apo data, *n* = 1697 adults aged 29 to 94 at the time of enrolment, were included in the study.

### Questionnaires and general demographics

Trained field workers used a standardised questionnaire developed for PURE to conduct an interview with volunteers regarding their demographics (age, locality and sex), health status, medication use, medical history and tobacco use [22]. T2DM, overweight, and obesity frequencies were determined based on glycated haemoglobin (HbA1C) and body mass index (BMI) levels using the World Health Organization cut-off points [25, 26]. A validated quantified food frequency questionnaire was used to determine the fat intake of the participants [27]. Suitable photographs of food portions were used to quantify the amounts consumed [28]. The reported food quantities were converted to kilojoules by using standardised Table [29]. An adapted BAECKE questionnaire was used to gather habitual physical activity data to determine a physical activity index value [30].

### Anthropometric measurements

Anthropometric measurements were done using standardised procedures from the International Society for the Advancement of Kinanthropometry (ISAK) [31]. Height was measured with an Invicta stadiometer (IP 1465, UK) and weight was measured with a precision health scale (A & D Company, Japan), after which BMI was calculated.

### Cardiovascular measurements

A validated automated digital OMRON HEM-757 device (Omron Healthcare, Kyoto, Japan) was used to measure office systolic and diastolic blood pressure according to the guidelines of the European Society of Hypertension and the European Society of Cardiology (ESH/ ESC) [32]. Measurements were performed using the brachial artery in the upper right arm that was supported at heart level in a sitting position. Two measurements were taken within a 5-minute interval, of which the second recorded measurement was used for data analysis [32].

### Biochemical analysis

HbA1C and human immunodeficiency virus (HIV) status were determined on-site using fasting blood samples. HbA1C levels were measured in ethylenediaminetetraacetic acid treated whole blood with an automated D-10 Haemoglobin testing system (Bio-Rad Laboratories, Hercules, CA). HIV status was assessed directly from whole blood using the First Response rapid HIV card test (Premier Medical Corporation Ltd, Daman, India), with positive results confirmed by the Pareeshak card test (BHAT Bio-tech, India). The remaining fasting serum samples were stored at -80 °C in temperature-monitored bio-freezers until further analysis. Serum levels of HDLC, TC, TG, γ-Glutamyl transferase (γGT) and C-reactive protein (CRP) were analysed with an automated Konelab20 analyser (Thermo Fisher Scientific Oy, Vantaa, Finland). LDLC levels were calculated by using the Friedewald formula [33]. The Apo multiplex magnetic bead immunoassays (Merck Millipore, Darmstadt, Germany) were conducted by using the serum samples diluted at 1:4000 and analysed with the Luminex 200 system (Luminex, Austin, TX, United States). Raw data were processed with the Belysa curve-fitting software (Merck Millipore, Darmstadt, Germany) to quantify the ApoA1 and ApoB levels.

### Statistical analysis

All statistical analyses were performed with IBM^®^ SPSS^®^ Statistics version 29 software (IBM Corporation; Armonk, New York, USA). Graphs were prepared with GraphPad Prism version 5.03 (GraphPad Software Inc., CA, USA). Q-Q plots were used to determine the distribution and normality of the data. Skewed variables (BMI, γGT, fat intake, TG, CRP and all Apos) were logarithmically transformed. CVD risk factors were profiled according to tertiles of the extended lipid profile using analysis of variance (ANOVA) for the continuous variables and Chi-square analysis for the categorical variables. Continuous data with a normal distribution were reported as the arithmetic mean and standard deviation, whereas the logarithmically transformed variables were reported by the geometric mean and 5th and 95th percentile intervals. Categorical data were presented as proportions. Analysis of covariance (ANCOVA) were performed with adjustments for age, sex and locality. To determine associations of conventional lipids and Apos (HDLC, LDLC, TC, TG, ApoA1, ApoB and ApoB: ApoA1) as dependent variables with modifiable CVD risk markers (BMI, physical activity index, tobacco use, dietary fat intake, γGT and HbA1C) as main independent variables, backwards multivariable linear regression models were used while adjusting for age, sex, locality, CRP, diuretic medication, HIV status and hypertension status. All model assumptions were assessed and met, including the normal distribution of residuals and homoscedasticity, which were confirmed through visual inspection of the residual plots. To avoid multicollinearity, separate regression analyses were conducted for each lipid and Apo marker.

## Results

Table 1 presents the population characteristics stratified by tertiles of the conventional lipid levels. In terms of the non-modifiable CVD risk markers, age was higher in the higher tertiles of LDLC, TC and TG (all *p* < 0.001). Urban locality was more prevalent in the higher tertiles of TG (*p* < 0.001) and male sex was more frequent in the higher tertiles of HDLC and more frequent in the lower tertiles of LDLC, TC and TG (all *p* < 0.001). When investigating differences in modifiable CVD risk markers, the highest tertiles of the atherogenic LDLC, TC and TG and the lowest tertiles for the anti-atherogenic HDLC, exhibited higher BMI and HbA1C levels (all *p* < 0.001). Notably, the highest tertiles of LDLC and TG contained the highest proportion of participants with T2DM, while the lowest tertile of HDLC also showed the highest prevalence of T2DM (all *p* ≤ 0.024). Similarly, overweight and obese participants were more prevalent in the highest tertiles of LDLC, TC and TG, as well as in the lowest tertiles of HDLC (all *p* < 0.001). Higher γGT levels were evident in the highest tertiles of all the conventional lipids (all *p* < 0.001), except for LDLC where no differences were found. Tobacco use was unexpectedly higher in the highest tertile of HDLC (*p* < 0.001), while being lower in the highest tertiles of LDLC (*p* < 0.001), TC (*p* = 0.006) and TG (*p* = 0.012). No differences for physical activity index and fat intake were observed across conventional lipid tertiles.

**Table 1:** Characteristics stratified by tertiles of conventional lipids

	Tertile 1	Tertile 2	Tertile 3	*p* trend
**High density lipoprotein cholesterol (mmol/L)**	< 1.18	1.18 ≥ to < 1.72	≥ 1.72	
n	568	566	563	
Age (years)	49.46 ± 10.43	50.56 ± 10.64	50.34 ± 9.83	0.175
Locality (Urban; %)	276 (48.59)	269 (47.53)	289 (51.33)	0.419
Sex (Men; %)	202 (35.56)^a^	193 (34.10)^b^	250 (44.40)^a, b^	**< 0.001**
Body mass index (kg/m^2^)	25.25 (16.87; 39.41)^a, c^	24.30 (16.68; 38.91)^b, c^	21.66 (15.70; 34.70)^a, b^	**< 0.001**
Physical activity index	7.31 ± 1.94	7.25 ± 1.77	7.27 ± 1.93	0.896
Tobacco use (use; %)	295 (51.94)^a^	295 (52.12)^b^	359 (63.77)^a, b^	**< 0.001**
Fat intake (kJ)	38.31 (12.82; 106.62)	39.70 (12.71; 115.71)	41.43 (12.37; 112.96)	0.159
γ-Glutamyl transferase (U/L)	45.07 (17.51; 239.87)^a^	49.96 (18.88; 339.28)^b^	80.04 (21.79; 469.25)^a, b^	**< 0.001**
Glycated haemoglobin (%)	5.71 (4.90; 6.85)^a^	5.64 (4.80; 6.60)^b^	5.44 (4.80; 6.10)^a, b^	**< 0.001**
C-reactive protein (mg/L)	4.12 (3.64; 4.66)^a, c^	3.07 (2.71; 3.47)^c^	2.58 (2.28; 2.91)^a^	**< 0.001**
Diuretic medication (use; %)	22 (3.87)	25 (4.42)	11 (1.95)	0.057
Human immunodeficiency virus status (positive; %)	155 (27.29)^a, c^	67 (11.84)^c^	59 (10.48)^a^	**< 0.001**
Hypertension status (hypertensive; %)	237 (41.73)^a^	262 (46.29)	302 (53.64)^a^	**< 0.001**
Type II diabetes mellitus status (diabetic; %)	47 (8.27)^a^	35 (6.19)^b^	10 (1.78)^a, b^	**< 0.001**
Adiposity status (Overweight and obese; %)	272 (48.06)^a^	244 (43.26)^b^	134 (23.80)^a, b^	**< 0.001**
**Low density lipoprotein cholesterol (mmol/L)**	< 2.31	≥ 2.31 to < 3.30	≥ 3.30	
n	566	565	566	
Age (years)	48.73 ± 9.27^a^	49.87 ± 10.42^b^	51.76 ± 10.97^a, b^	**< 0.001**
Locality (Urban; %)	276 (48.76)	276 (48.85)	282 (49.82)	0.925
Sex (Men; %)	282 (49.82)^a, c^	201 (35.58)^b, c^	162 (28.62)^a, b^	**< 0.001**
Body mass index (kg/m^2^)	21.87 (15.76; 36.22)^a, c^	23.60 (16.24; 38.95)^b, c^	25.76 (17.81; 39.89)^a, b^	**< 0.001**
Physical activity index	7.28 ± 1.86	7.32 ± 2.01	7.24 ± 1.76	0.765
Tobacco use (use; %)	358 (63.25)^a, c^	306 (54.16)^c^	285 (50.35)^a^	**< 0.001**
Fat intake (kJ)	38.35 (11.41; 114.63)	40.11 (15.28; 105.99)	40.94 (13.01; 113.67)	0.280
γ-Glutamyl transferase (U/L)	60.56 (18.32; 407.55)	54.59 (19.00; 379.59)	54.40 (19.32; 303.85)	0.085
Glycated haemoglobin (%)	5.46 (4.70; 6.30)^a, c^	5.62 (4.90; 6.67)^c^	5.70 (4.90; 6.76)^a^	**< 0.001**
C-reactive protein (mg/L)	3.08 (2.70; 3.51)	2.84 (2.51; 3.22)^b^	3.73 (3.33; 4.18) ^b^	**0.005**
Diuretic medication (use; %)	12 (2.12)^a^	18 (3.19)	28 (4.95)^a^	**0.030**
Human immunodeficiency virus status (positive; %)	114 (20.14)^a^	110 (19.47)^b^	57 (10.07)^a, b^	**< 0.001**
Hypertension status (hypertensive; %)	251 (44.35)	264 (46.73)	286 (50.53)	0.090
Type II diabetes mellitus status (diabetic; %)	19 (3.36)^a^	34 (6.03)	39 (6.89)^a^	**0.024**
Adiposity status (Overweight and obese; %)	146 (25.80)^a, c^	212 (37.59)^b, c^	292 (51.77)^a, b^	**< 0.001**
**Total cholesterol (mmol/L)**	< 4.32	≥4.32 to < 5.48	≥ 5.48	
n	566	564	567	
Age (years)	48.10 ± 9.57^a, c^	50.43 ± 10.34^c^	51.83 ± 10.67^a^	**< 0.001**
Locality (Urban; %)	268 (47.35)	274 (48.58)	292 (51.50)	0.357
Sex (Men; %)	259 (45.76)^a, c^	208 (36.88)^c^	178 (31.39)^a^	**< 0.001**
Body mass index (kg/m^2^)	22.54 (16.01; 37.40)^a, c^	23.62 (16.23; 37.46)^b, c^	24.97 (17.24; 39.27)^a, b^	**< 0.001**
Physical activity index	7.32 ± 1.92	7.29 ± 1.92	7.22 ± 1.81	0.610
Tobacco use (use; %)	344 (60.78)^a^	313 (55.50)	292 (51.50)^a^	**0.006**
Fat intake (kJ)	38.33 (11.87; 113.10)	39.48 (12.76; 109.31)	41.61 (13.17; 112.47)	0.123
γ-Glutamyl transferase (U/L)	49.19 (17.46; 325.43) ^a, c^	57.02 (19.24; 373.89)^c^	64.11 (21.02; 379.11)^a^	**< 0.001**
Glycated haemoglobin (%)	5.49 (4.80; 6.30)^a, c^	5.61 (4.80; 6.50)^c^	5.68 (4.90; 6.86)^a^	**< 0.001**
C-reactive protein (mg/L)	3.16 (2.76; 3.61)	2.96 (2.62; 3.34)	3.49 (3.12; 3.92)	0.142
Diuretic medication (use; %)	9 (1.59)^a, c^	24 (4.26)^c^	25 (4.41)^a^	**0.013**
Human immunodeficiency virus status (positive; %)	145 (25.62)^a, c^	86 (15.25)^b, c^	50 (8.82)^a, b^	**< 0.001**
Hypertension status (hypertensive; %)	226 (39.93)^a, c^	270 (47.87)^c^	305 (53.79)^a^	**< 0.001**
Type II diabetes mellitus status (diabetic; %)	21 (3.71)	33 (5.86)	38 (6.70)	0.072
Adiposity status (Overweight and obese; %)	173 (30.62)^a, c^	212 (38.26)^b, c^	262 (46.29)^a, b^	**< 0.001**
**Triglyceride (mmol/L)**	< 0.89	≥ 0.89 to < 1.38	≥ 1.38	
n	571	574	552	
Age (years)	48.15 ± 9.89^a, c^	50.21 ± 10.32^b, c^	52.06 ± 10.38^a, b^	**< 0.001**
Locality (Urban; %)	269 (47.11)^a^	253 (44.08)^b^	312 (56.52)^a, b^	**< 0.001**
Sex (Men; %)	264 (46.23)^a, c^	210 (36.59)^c^	171 (30.98)^a^	**< 0.001**
Body mass index (kg/m^2^)	22.05 (16.17; 34.54)^a, c^	23.25 (15.82; 37.34)^b, c^	26.01 (17.83; 41.10)^a, b^	**< 0.001**
Physical activity index	7.38 ± 1.79	7.25 ± 1.88	7.20 ± 1.97	0.249
Tobacco use (use; %)	344 (60.24)^a^	322 (56.10)	283 (51.27)^a^	**0.012**
Fat intake (kJ)	38.73 (12.69; 109.64)	38.76 (11.99; 108.52)	42.05 (13.22; 115.28)	0.074
γ-Glutamyl transferase (U/L)	51.30 (17.42; 380.31)^a^	53.72 (19.00; 339.03)^b^	65.63 (20.72; 380.04)^a, b^	**< 0.001**
Glycated haemoglobin (%)	5.46 (4.80; 6.10)^a, c^	5.54 (4.77; 6.40)^b, c^	5.80 (4.90; 7.70)^a, b^	**< 0.001**
C-reactive protein (mg/L)	2.56 (2.25; 2.90)^a, c^	3.32 (2.93; 3.76)^c^	3.88 (3.45; 4.36)^a^	**< 0.001**
Diuretic medication (use; %)	8 (1.40)^a^	18 (3.14)	32 (5.80)^a^	**< 0.001**
Human immunodeficiency virus status (positive; %)	93 (16.29)	107 (18.64)	81 (14.67)	0.187
Hypertension status (hypertensive; %)	235 (41.16)^a^	251 (43.73)^b^	315 (57.07)^a, b^	**< 0.001**
Type II diabetes mellitus status (diabetic; %)	11 (1.93)^a^	23 (4.01)^b^	58 (10.50)^a, b^	**< 0.001**
Adiposity status (Overweight and obese; %)	154 (27.07)^a, c^	206 (35.89)^b, c^	290 (52.73)^a, b^	**< 0.001**

Unadjusted results are presented as arithmetic mean ± standard deviation, geometric mean with 5th and 95th percentile intervals, or frequency and percentage

Superscript letters indicate significance for between tertile differences

*Abbreviations*: n, number of participants; CVD, cardiovascular disease

**Table 2:** Characteristics stratified by tertiles of apolipoproteins

	Tertile 1	Tertile 2	Tertile 3	*p* trend
**Apolipoprotein A1 (mg/dL)**	< 63.10	≥ 63.10 to < 85.11	≥ 85.11	
n	564	556	577	
Age (years)	49.92 ± 10.59	50.72 ± 10.54	49.74 ± 9.80	0.236
Locality (Urban; %)	261 (46.28)^a^	264 (47.48)	309 (53.55)^a^	**0.031**
Sex (Men; %)	204 (36.17)	208 (37.41)	233 (40.38)	0.321
Body mass index (kg/m^2^)	24.58 (16.46; 38.67)^a^	24.04 (16.73; 39.80)^b^	22.53 (16.12; 35.75)^a, b^	**< 0.001**
Physical activity index	7.33 ± 1.87	7.24 ± 1.92	7.26 ± 1.86	0.690
Tobacco use (use; %)	298 (52.84)^a^	293 (52.70)^b^	358 (62.05)^a, b^	**0.002**
Fat intake (kJ)	38.55 (13.14; 109.09)	39.90 (13.74; 110.39)	40.95 (11.73; 115.63)	0.328
γ-Glutamyl transferase (U/L)	43.32 (17.79; 185.50)^a, c^	55.72 (19.90; 351.38)^b, c^	74.01 (19.74; 440.30)^a, b^	**< 0.001**
Glycated haemoglobin (%)	5.70 (4.90; 6.97)^a, c^	5.59 (4.90; 6.50)^b, c^	5.49 (4.80; 6.30)^a, b^	**< 0.001**
C-reactive protein (mg/L)	3.57 (3.15; 4.04)	3.24 (2.85; 3.67)^b^	2.84 (2.52; 3.20)^b^	**0.035**
Diuretic medication (use; %)	14 (2.48)	26 (4.68)	18 (3.12)	0.115
Human immunodeficiency virus status (positive; %)	137 (24.29)^a, c^	75 (13.49)^c^	69 (11.96)^a^	**< 0.001**
Hypertension status (hypertensive; %)	239 (42.38)^a^	261 (46.94)	301 (52.17)^a^	**0.005**
Type II diabetes mellitus status (diabetic; %)	47 (8.33)^a^	29 (5.22)	16 (2.78)^a^	**< 0.001**
Adiposity status (Overweight and obese; %)	245 (43.44)^a^	226 (40.72)^b^	179 (31.18)^a, b^	**< 0.001**
**Apolipoprotein B (mg/dL)**	< 64.57	≥ 64.57 to < 91.20	≥ 91.20	
n	573	545	579	
Age (years)	50.36 ± 10.15	49.88 ± 9.98	50.11 ± 10.79	0.734
Locality (Urban; %)	299 (52.18)	261 (47.89)	274 (47.32)	0.199
Sex (Men; %)	260 (45.38)^a, c^	179 (32.84)^c^	206 (35.58)^a^	**< 0.001**
Body mass index (kg/m^2^)	22.27 (15.75; 36.74)^a, c^	24.10 (16.55; 38.95)^c^	24.77 (16.94; 38.57)^a^	**< 0.001**
Physical activity index	7.15 ± 1.97	7.32 ± 1.81	7.37 ± 1.85	0.130
Tobacco use (use; %)	355 (61.95)^a, c^	294 (53.94)^c^	300 (51.81)^a^	**0.001**
Fat intake (kJ)	40.22 (12.64; 109.60)	38.85 (12.81; 110.61)	40.30 (11.80; 117.00)	0.591
γ-Glutamyl transferase (U/L)	59.17 (18.33; 385.20)	54.29 (19.25; 334.35)	55.89 (18.94; 378.32)	0.270
Glycated haemoglobin (%)	5.47 (4.77; 6.30) ^a, c^	5.65 (4.90; 6.74)^c^	5.66 (4.80; 6.70)^a^	**< 0.001**
C-reactive protein (mg/L)	3.09 (2.72; 3.52)	3.26 (2.87; 3.69)	3.24 (2.88; 3.65)	0.821
Diuretic medication (use; %)	11 (1.91)^a^	19 (3.49)	28 (4.83)^a^	**0.024**
Human immunodeficiency virus status (positive; %)	87 (15.18)	101 (18.53)	93 (16.06)	0.295
Hypertension status (hypertensive; %)	278 (48.52)	253 (46.42)	270 (46.63)	0.773
Type II diabetes mellitus status (diabetic; %)	19 (3.32)^a, c^	36 (6.61)^c^	37 (6.40)^a^	**0.023**
Adiposity status (Overweight and obese; %)	165 (28.80)^a, c^	222 (40.88)^c^	263 (45.58)^a^	**< 0.001**
**Apolipoprotein B: Apolipoprotein A1**	< 0.85	≥ 0.85 to < 1.26	≥ 1.26	
n	559	583	555	
Age (years)	49.19 ± 9.29 ^a^	50.25 ± 10.74	50.92 ± 10.77^a^	**0.014**
Locality (Urban; %)	307 (54.92)^a^	292 (50.09)^b^	235 (42.34)^a, b^	**< 0.001**
Sex (Men; %)	256 (45.80)^a, c^	199 (34.13)^c^	190 (34.23)^a^	**< 0.001**
Body mass index (kg/m^2^)	21.50 (15.56; 35.54)^a, c^	24.08 (16.76; 36.85)^b, c^	25.67 (17.25; 40.29)^a, b^	**< 0.001**
Physical activity index	7.21 ± 1.96	7.27 ± 1.95	7.35 ± 1.72	0.488
Tobacco use (use; %)	360 (64.40)^a, c^	312 (53.52)^c^	277 (49.91)^a^	**< 0.001**
Fat intake (kJ)	41.91 (12.41; 115.63) ^a^	40.21 (13.46; 114.43)	37.37 (12.05; 104.90)^a^	**0.018**
γ-Glutamyl transferase (U/L)	71.14 (19.65; 452.40)^a, c^	54.01 (18.63; 357.36)^b, c^	46.85 (18.99; 222.90)^a, b^	**< 0.001**
Glycated haemoglobin (%)	5.42 (4.70; 6.10)^a, c^	5.60 (4.90; 6.50)^b, c^	5.76 (4.90; 7.12)^a, b^	**< 0.001**
C-reactive protein (mg/L)	2.81 (2.48; 3.18)^a^	2.84 (2.52; 3.21)^b^	4.13 (3.65; 4.67)^a, b^	**< 0.001**
Diuretic medication (use; %)	9 (1.61)^a^	21 (3.60)	28 (5.05)^a^	**0.007**
Human immunodeficiency virus status (positive; %)	69 (12.34)^a^	94 (16.12)	118 (21.26)^a^	**< 0.001**
Hypertension status (hypertensive; %)	282 (50.45)	276 (47.34)	243 (43.78)	0.105
Type II diabetes mellitus status (diabetic; %)	11 (1.97)^a, c^	33 (5.67)^c^	48 (8.65)^a^	**< 0.001**
Adiposity status (Overweight and obese; %)	130 (37.70)^a, c^	236 (39.31)^b.c^	284 (38.21)^a, b^	**< 0.001**

Unadjusted results are presented as arithmetic mean ± standard deviation, geometric mean with 5th and 95th intervals, or frequency and percentage

Superscript letters indicate significance for between tertile differences

*Abbreviations*: n, number of participants; CVD, cardiovascular disease

When profiling CVD risk factors across tertiles of Apo levels (Table 2), similar patterns as for the conventional lipids were observed. Among the non-modifiable CVD risk factors, age was higher in the higher tertiles of ApoA1:ApoB (*p* = 0.014). Urban locality was more prevalent in the higher tertiles of ApoA1 (*p* = 0.031), but less prevalent in the higher tertiles of ApoA1:ApoB (*p* < 0.001). Male sex was less frequent in the higher tertiles of ApoB and ApoA1:ApoB (*p* < 0.001). In terms of the modifiable CVD risk markers, BMI and HbA1C levels were higher in the highest tertiles of the atherogenic ApoB and ApoB: ApoA1 (all *p* < 0.001), with the opposite found with the anti-atherogenic ApoA1 (all *p* < 0.001). The highest tertiles of ApoB and ApoB: ApoA1 included the largest proportion of participants with T2DM and those classified as overweight or obese (all *p* ≤ 0.023). The lowest tertiles of ApoA1 also showed the highest proportions of the overweight and T2DM participants (all *p* < 0.001). In contrast, the prevalence of tobacco use was lower in the highest tertiles of the atherogenic ApoB and ApoB: ApoA1 (all *p* < 0.001) and higher in the highest tertiles of the anti-atherogenic ApoA1 (all *p* = 0.002). Fat intake displayed lower levels in the highest ApoB: ApoA1 tertile (*p* = 0.018). For γGT, lower levels were found in the highest tertiles of the atherogenic ApoB: ApoA1 (*p* < 0.001) and higher levels in the highest tertiles of the anti-atherogenic ApoA1 (*p* < 0.001).

**Fig. 1:**
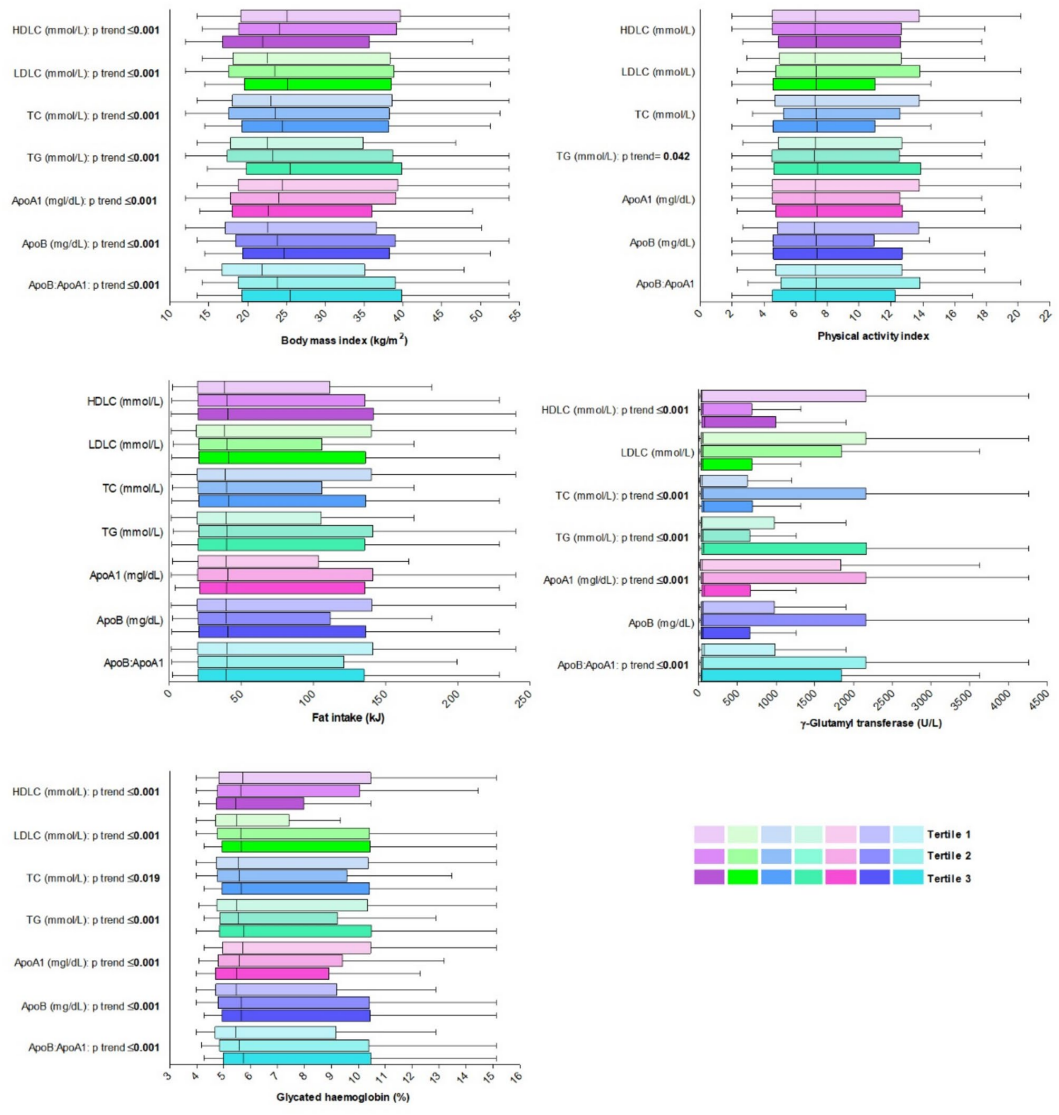
Adjusted differences in CVD risk markers stratified by tertiles of conventional lipids and apolipoproteins. The boxplots show the adjusted mean values, minimum and maximum values and the lower and upper bounds of the 95% confidence intervals. The modifiable CVD risk markers are divided into tertiles for each conventional lipid and Apo marker. The analysis is adjusted for age, locality and sex. Only significant p trend values are given

Differences in the CVD risk factors, after adjusting for age, locality and sex are presented in Fig. 1. The findings align closely with the previously described unadjusted findings, except for the physical activity index (*p* = 0.042) now being greater in the highest TG tertile.

**Fig. 2:**
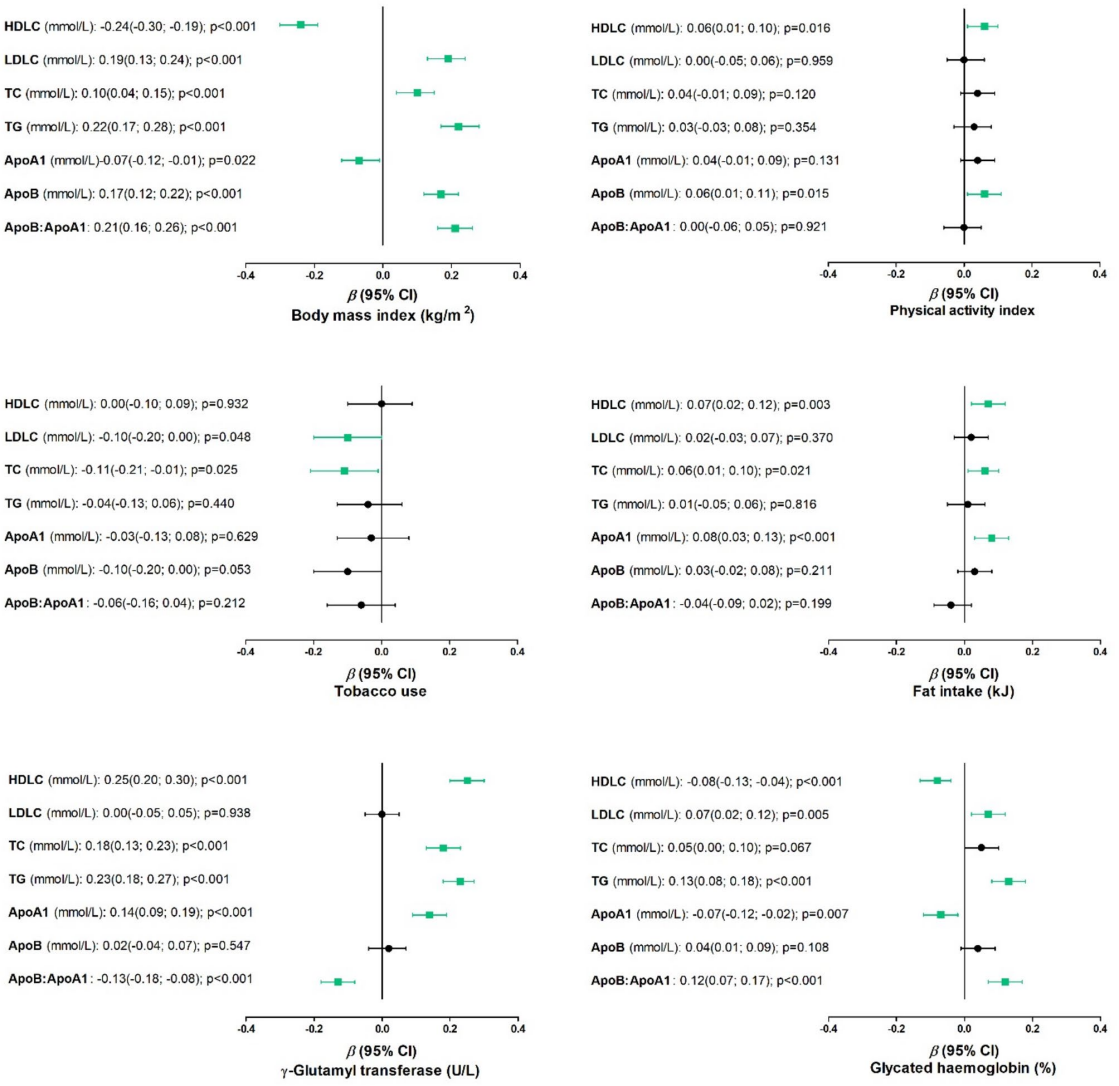
Multivariable linear adjusted associations of conventional lipids, apolipoproteins and cardiovascular disease risk factors. Multivariable linear regression models of each conventional lipid and Apo marker, categorised by modifiable risk factors, are adjusted for age, CRP, use of diuretics, HIV status, hypertension status, locality, and sex. The boxplots display significant β-values with a green square compared to insignificant values displayed with a black circle. The boxplots were further structured using the lower and upper bounds of the 95% confidence intervals

The adjusted associations of the conventional lipids, Apos and CVD risk factors are shown in Fig. 2. Among the conventional lipids, negative associations for the anti-atherogenic HDLC and positive associations for the atherogenic LDLC, TC, and TG were found with BMI (all *p* < 0.001) and HbA1C (all *p* ≤ 0.067). Positive associations were found between HDLC, TC and TG with γGT (all *p* < 0.001), while negative associations were found between LDLC and TC with tobacco use (both *p* ≤ 0.048). Only a few associations between all lipid markers and PA as well as fat intake were observed. A positive association between HDLC and PA was seen (*p* = 0.016), while HDLC and TC was positively associated with fat intake (both *p* ≤ 0.021).

In terms of the Apos, the anti-atherogenic ApoA1 was negatively associated with BMI (β=-0.067 (-0.125; -0.010), *p* = 0.022) and HbA1C (β=-0.071 (-0.122; -0.020), *p* = 0.007). The atherogenic ApoB, as well as ApoB: ApoA1, was positively associated with BMI (ApoB: β = 0.168 (0.117; 0.218), *p* < 0.001 and ApoB: ApoA1: β = 2.13 (0.163; 0.263), *p* < 0.001), while ApoB was also positively associated with HbA1C (β = 0.123 (0.074; 0.172), *p* < 0.001). γGT associated positively with ApoA1 (β = 0.135 (0.084; 0.185), *p* < 0.001) and negatively with ApoB: ApoA1 (β=-0.129 (-0.177; -0.081), *p* < 0.001). Positive associations between ApoB and physical activity (β = 0.060 (0.012; 0.108), *p* = 0.015) and ApoA1 and fat intake (β = 0.081 (0.033; 0.130), *p* < 0.001) were found. No Apo marker was associated with tobacco use.

## Discussion

Our most prominent finding was that the Apo markers associated with the CVD risk markers and reflected similar atherogenic patterns to those of conventional lipids among South African adults of African ancestry. This supports previous evidence on the potential of ApoA1, ApoB and ApoB: ApoA1 to predict CVD risk, as has also been observed in Asian and European populations [15–17].

When examining the differences in CVD risk markers stratified by tertiles of the conventional lipid and Apo markers, various unadjusted trends emerged for BMI, HbA1C, γGT and tobacco use. Several investigated potential confounders showed expected trends with higher atherogenic and lower anti-atherogenic lipids and Apos with older age [34], urban locality [34], increased CRP levels [35], diuretic use [36], HIV positive status [37] and hypertension [36]. The observed trend with CRP levels further suggests the atherosclerosis mechanism at play as CRP is a well-established acute phase reactant and biomarker of systemic inflammation, which contributes to the initiation, progression, and destabilisation of atherosclerotic plaques and ultimately CVD [38]. Male sex also aligned consistent with results reported from a previous study among South African adults with lower atherogenic and higher anti-atherogenic lipids levels for men [39].

Similar associations were observed between conventional lipid and apolipoprotein markers and CVD risk markers after adjusting for the confounders in regression analyses. Both BMI and HbA1c were positively associated with all atherogenic lipids and Apos and negatively associated with all anti-atherogenic ones. The link between BMI, HbA1C and CVD risk through the atherosclerotic process is well documented [13]. Obesity, characterised by adipocyte hypertrophy, proliferation and adipose tissue expansion, leads to increased lipid storage and thus contributes to adverse lipid metabolism [40]. This, in turn, exacerbates CVD risk factors such as high blood pressure, hyperglycaemia and inflammation [40], where chronic inflammation further accelerates atherosclerosis, compounding these risks [41]. The increased presence of circulating atherogenic lipoproteins fosters the development of cholesterol plaques in arterial walls, potentially leading to severe atherosclerotic cardiovascular events like myocardial infarction and stroke [40].

Hyperglycaemia itself impacts CVD risk by inducing dyslipidaemia, characterised by elevated LDLC, TG, ApoB and decreased HDLC levels [42, 43]. Dyslipidaemia caused by hyperglycaemia is linked to impaired liver Apo production, which affects lipoprotein lipase and cholesterol ester transport protein activities, leading to atherogenic changes [44]. Hyperglycaemia also generates reactive oxygen species and increase lipid peroxidation [44]. This oxidative stress results in non-enzymatic glycosylation of low density lipoproteins, which enhances their incorporation into arterial walls and decreases its metabolism due to impaired low density lipoprotein receptor interactions [44]. Consequently, also contributing to early onset atherosclerosis and increased cardiovascular complications [44].

In addition to the findings regarding BMI and HbA1c, our study revealed expected and unexpected associations with γGT. γGT is involved in glutathione uptake, oxidative stress, inflammation and is considered a general biomarker of alcohol misuse [45]. We found that γGT was positively associated with TC, TG, and HDLC, as well as with ApoA1, which aligns with prior research conducted among White and Bangladeshi adults [20, 45]. In contrast, we observed a negative association between γGT and ApoB: ApoA1, which is inconsistent with earlier studies conducted in Pakistan, Italy and China [46–48]. This unexpected result may indicate a protective mechanism against atherosclerosis, as suggested by the negative ratio of atherogenic to anti-atherogenic Apos, a phenomenon which was previously reported among Black South Africans [49]. Further, the relationship between γGT and conventional lipid and Apo markers could vary depending on other underlying health factors such as non-alcoholic fatty liver disease and systemic inflammation [50].

Of all the CVD risk factors investigated, tobacco use appears to relate to none of the Apos. Surprisingly, we showed that atherogenic conventional lipids (TC and LDLC) associated with a lower prevalence of tobacco use. It was previously suggested that the seemingly favourable conventional lipid profile observed in smokers might be due to the increased energy expenditure, reduced appetite, and weight loss associated with smoking [51]. It is also important to note that tobacco usage was self-reported by the participants for this study and cotinine analysis might provide a more accurate representation of the risk factor occurrence.

Only a few associations were found between physical activity and conventional lipid and Apo markers, with physical activity positively associated with both HDLC and ApoB levels, presenting a contradicting atherogenic profile. The ApoB: ApoA1 marker, which could provide insight into the atherogenic balance, did not show a significant association. Increased HDLC levels align with the expected pronounced relationship of HDLC and physical activity [52]. Similarly, dietary fat intake showed limited but notable associations with conventional lipid and Apo markers. Higher dietary fat intake was linked to higher levels of HDLC, TC and ApoA1. The positive association with HDLC reflected in anti-atherogenic ApoA1 levels.

Our study has several notable strengths, including a substantial sample size, the use of validated questionnaires and the incorporation of biochemical data. The biochemical data obtained through single analyses could be repeated for improved robustness and cotinine analysis could perhaps provide a more precise assessment of tobacco use. To our knowledge, this is the first study of this scale to examine the association of multiple CVD risk factors with conventional lipids and Apos in South African adults of African ancestry. This was a cross-sectional analysis and future studies should use a longitudinal design to determine the potential of Apos to predict CVD development.

## Conclusion

Our findings in South African adults of African ancestry indicate that ApoA1, ApoB and ApoB: ApoA1 levels exhibit atherogenic patterns similar to those of conventional lipids when linked to established CVD risk markers. This highlights the potential of these apolipoprotein markers to offer deeper insights into the atherogenic cascade, going beyond the conventional lipid profile to explain the development of early cardiometabolic disease and, ultimately, the progression of CVD.

## Data Availability

The data that support the findings of this study are available from the corresponding author, but restrictions apply to the availability of these data, which were used under approval of Health Research Ethics Committee of the North-West University Potchefstroom, South Africa for the current study, and so are not publicly available.

## Abbreviations

HDLC: High density lipoprotein cholesterol
LDLC: Low density lipoprotein cholesterol
TC: Total cholesterol
TG: Triglycerides
CVD: Cardiovascular disease
Apo: Apolipoprotein
ApoB: ApoA1:Apolipoprotein B to Apolipoprotein A1 ratio
PURE: Prospective Urban Rural Epidemiology
BMI: Body mass index
γGT: γ-Glutamyl transferase
HbA1C: Glycated haemoglobin
T2DM: Type II diabetes mellitus
ISAK: International Society for the Advancement of Kinanthropometry
ESH/ESC: European Society of Hypertension/European Society of Cardiology
HIV: Human immunodeficiency virus
ANOVA: Analysis of Variance
ANCOVA: Analysis of Covariance
IBM® SPSS®: International Business Machines Statistical Package for the Social Sciences
n: Number of participants
